# General Considerations—Static Electricity

**Published:** 1913-04-12

**Authors:** Alfred C. Norman

**Affiliations:** House Surgeon at Durham County Eye Infirmary.


### ELECTRICITY IN MODERN MEDICINE.*

### XXVII. General Considerations—Static Electricity.

By ALFRED C. NORMAN, M.D. Edin., House Surgeon at Durham County Eye Infirmary.

Before going on to a consideration of static electricity a little more remains to be said about Leduc currents and about the action of galvanic and faradic currents upon the human body.

*Leduc's interrupted galvanic currents* present obvious advantages over currents obtained from faradic coils for research and for accurate electro-diagnosis. It has been shown that the discharge from faradic coils is liable to considerable variation as regards regularity, rate of interruption, duration of impulse, ratio of impulse to interval, and voltage. Further, these factors are very little under our control and direct measurement of the current is quite out of the question. Obviously, then, a report on the "faradic" reaction of a muscle made with the aid of a medical coil is purely empirical and conveys but little information to anyone who is unacquainted with the particular coil used. On the other hand, a report made with a Leduc interrupter presents a universal basis of comparison between the normal and the abnormal, for all the above factors can be directly controlled and the strength of current accurately measured.

An ideal outfit for muscle testing would consist of an ordinary dry-cell galvanic battery with a milliampere-meter, rheostat, and electrodes for estimating galvanic reactions, with the addition of a Leduc interrupter for the estimation of faradic reactions, the motor of the latter being driven from an accumulator or the street mains.

It must not be supposed, however, that the faradic battery is a useless piece of apparatus. It is exceedingly valuable in simple electrical treatment where stimulation of skin, muscle, and nerve is required.

*Electric Sleep.*—By means of his interrupted galvanic currents Leduc has induced a form of unconsciousness ("electric sleep") and partial anæsthesia in animals and has even allowed it to be tried on himself. A large electrode, connected with the negative pole of the apparatus, is placed on the patient's forehead while the positive electrode is strapped to his back. A current of about 4 milliamperes at 35 volts, interrupted about 100 times per second by the Leduc apparatus, is passed through the patient in order to obtain the desired effect. Unconsciousness and anæsthesia are not very profound, induction is a protracted process, and the sensations of the patient in going under are said to resemble a nightmare, hence it is problematical whether the method will ever be universally adopted, but it is at any rate of extreme scientific interest.

### The Action of Galvanic and Faradic Currents upon the Human Body.

A full description of the action of electricity upon healthy and diseased muscle and nerve can be obtained from text-books of physiology and nervous diseases respectively, but a few practical points, having a direct bearing on muscle testing and the reaction of degeneration, will be given here:—

(1) When a muscle and its nerve are both healthy an electrical current produces a contraction as a result of a stimulus passing *through the nerve* and its terminations. This applies to both galvanic and faradic currents, and the greatest response to a current of given strength takes place when the active electrode is applied over the "motor-point," *i.e.* the point at which the main nerve trunk enters the muscle.

(2) *A galvanic current* produces a muscular contraction only at the moment when the current is turned on or off, or suddenly increased or diminished in strength, or when it is reversed. *No contraction takes place while the current is flowing steadily.* Muscle testing with the galvanic current consists in noting the minimum current that will produce a contraction, and the nature of that contraction, when the current is turned on (closing contraction) and when it is turned off (opening contraction), first with the active electrode positive (anodal contraction) and secondly with it negative (cathodal contraction).

To facilitate these observations the active electrode should have a switch (contact maker) in the handle and there should be a current reverser connected with the battery.

A galvanic current that is made and broken more than 20 times per second throws the muscle into a state of tetanus, *i.e.* the muscle has not time to relax from one contraction before the next stimulus is upon it.

A galvanic current (provided it be not rapidly made and broken) will act directly upon the muscle even if the nerve be degenerated, but the type of contraction is quite different from that produced as a result of stimulation through the nerve.

(3) *A faradic current* throws the muscle into a state of tetanus during the whole time it is applied. In other words, its action is no more than that of a rapidly interrupted galvanic current.

Faradic currents will not produce a contraction in a muscle when the motor-nerve is degenerated.

### Reaction of Degeneration.

Reaction of degeneration occurs when a motor-nerve becomes degenerated in its lower neurone. The phenomena manifest themselves in from seven to fourteen days after injury and are easily explained in the light of the above generalisations. They are as follows:—

(1) The muscle does not contract to faradic currents—whether they be true faradic, sinusoidal, or Leduc currents.

* Previous articles appeared on Nov. 11, 25. Dec. 9. 30. 1911; Jan. 13, 27, Feb. 17, March 9, 30, April 20, May 4, 25, June 8, July 6, Aug. 3, 17, 31, Sept. 28, Oct. 12, 26, Nov. 9, 30, Dec. 14, 1912; Jan. 11, Feb. 1, and March 1.

(2) The contraction produced by a galvanic current is considerably altered in character owing to the fact that the stimulus takes place directly through the muscle itself and is conducted along it from fibre to fibre. The resulting contraction is a lethargic wavelike contraction that spreads slowly along the muscle and is particularly well brought out if the electrode be applied to the distal end of the muscle.

This lethargic contraction to galvanic currents is characteristic of reaction of degeneration, and when first established it can be elicited by a weaker current than the minimal necessary to stimulate a normal muscle. As time goes on, however, the muscle becomes less easily excited and requires a current stronger than normal to produce any response.

(3) Normally with a given strength of galvanic current the closing contraction (*i.e.* the contraction that takes place when the current is switched on) is greater when the active electrode is the *cathode* than when it is the *anode*, but usually in reaction of degeneration the anode excites the muscle more than the cathode at closing—in other words, A.C.C. becomes greater than C.C.C. This phenomenon is of considerably less importance than Nos. 1 and 2, however, and the beginner need not worry himself to elicit it if the latter are quite definite, for they are sufficient in themselves to establish a diagnosis of reaction of degeneration.

### Static Electricity.

The discovery, if discovery it may be called, of electricity dates back to 600 B.C., when Thales of Miletus, the chief of the seven wise men of ancient Greece, recorded the fact that amber after having been rubbed would attract various light bodies. It is remarkable that further investigation of this property was not undertaken until the year 1600, when Gilbert of Colchester found that many other substances possessed the same property, and these he termed "electrics" from the Greek "elektron," amber. And it is still more remarkable that other ways of producing what we now call "electricity" were not discovered until the end of the eighteenth century.

Static electricity is electricity at rest. If we rub a glass rod with a piece of fur the rod becomes negatively and the fur positively charged with electricity. In other words, if we imagine that rod and fur were both originally possessed of a certain amount of electricity, the fur now contains more and the rod less than its share, as a result of the work done in the application of friction. There is now a difference of electrical pressure between the two substances, and the electricity on their surfaces only remains at rest because they are bad conductors. If the surfaces of the rod and the fur were connected by means of a metallic conductor there would be a flow of electricity between them that would continue until it was once more equally distributed throughout both. Friction between any two dissimilar substances separates a certain amount of electricity, but when they are good conductors it is immediately dispersed unless precautions are taken to insulate the conductors. Because they neglected to do this the ancients were for many centuries under the impression that only non-conductors were "electrics." The earliest forms of electrical machines were all frictional static machines. A ball of sulphur, or a glass cylinder, or, in the later machines, a glass plate, was caused to revolve against some substance capable of producing friction, and the feeble charge so obtained was applied to the patient.

The modern static machine used in electro-therapeutics depends upon the principle of induction. The outfit is elaborate and costly, takes up a good deal of room, and, moreover, in the damp climate of this country is liable to get out of order and give a lot of trouble—hence its application is best left in the hands of the specialist, and a full description would be out of place in a series of articles such as these. In America the Holtz machine is largely used, in this country the Wimshurst.

The Wimshurst machine, briefly described, consists of from eight to twelve circular plates of glass (or they may be of ebonite) with metal sectors, mounted on a horizontal axle in such a way that alternate plates revolve in opposite directions. When these plates are revolved at a high rate of speed (by some mechanical means such as an electric or water motor) they have an inductive influence upon each other, and an electric charge is generated which is collected by metallic combs and conveyed to two large brass knobs on the top of the machine known as the *prime conductors*. While the machine is working, one of these knobs is always positively and the other negatively charged to a high voltage, the available amperage being very small. If the knobs are brought near enough together the charge ceases to be "static" and jumps across the air-gap in a series of sparks, but the flow of current is always unidirectional because the knobs are always unipolar.

An insulated patient connected with one or other pole of a static machine can be charged up to his full capacity with either positive or negative electricity of high voltage, and will remain so charged with "electricity at rest" as long as he is connected with the machine and remains insulated. The induction coil could not be used to charge a patient with *static* electricity because its discharging pillars become alternately positive and negative. Hence an insulated patient connected with one pillar of the coil would receive an oscillatory discharge, not a static charge.

Of course, if a patient were connected with both poles of a static machine he would receive a perfectly unidirectional current through his body. This is never done in the case of a patient but in America, where climatic conditions are more suitable; immense static machines are often used to excite $x$-ray tubes and the unidirectional nature of the discharge is very advantageous for this purpose.

